# Effects of plyometric jump training on measures of physical fitness and lower-limb asymmetries in prepubertal male soccer players: a randomized controlled trial

**DOI:** 10.1186/s13102-024-00821-9

**Published:** 2024-02-06

**Authors:** Senda Sammoud, Yassine Negra, Raja Bouguezzi, Rodrigo Ramirez-Campillo, Jason Moran, Chris Bishop, Helmi Chaabene

**Affiliations:** 1 Research Laboratory (LR23JS01) “Sport Performance, Health and Society”, University “La Manouba”, 2037 Manouba, Tunisia; 2 Higher Institute of Sport and Physical Education of Ksar Saïd, University “La Manouba”, 2037 Manouba, Tunisia; 3 Exercise and Rehabilitation Sciences Institute. School of Physical Therapy, Faculty of Rehabilitation Sciences, Universidad Andres Bello, 7591538 Santiago, Chile; 4 School of Sport, Rehabilitation and Exercise Sciences, University of Essex, Colchester, Essex UK; 5 Faculty of Science and Technology, London Sports Institute, StoneX Stadium, Greenlands Lane, London, UK; 6 Division of Training and Movement Sciences, Research Focus Cognition Sciences, University of Potsdam, Am Neuen Palais 10, Building 12, 14469 Potsdam, Germany; 7 High Institute of Sports and Physical Education, University of Jendouba, 8189 Kef, Tunisia

**Keywords:** Football, Stretch–shortening cycle exercise, Human physical conditioning, Jumping ability, Youth sports

## Abstract

**Background:**

High level of physical fitness is a paramount soccer performance factor. As such, developing key components of physical fitness such as sprinting, jumping, and change of direction (CoD) at an early age empowers both short- and long-term performance success. Although previous research in prepubertal male soccer players has reported physical fitness performance enhancements following plyometric jump training (PJT), the effects on inter-limb asymmetries remain unclear.

**Objective:**

To assess the effects of PJT on measures of physical fitness and inter-limb asymmetries in prepubertal male soccer players.

**Methods:**

A total of 27 participants were recruited, and randomly assigned to either a PJT group (n = 13; age = 12.7 ± 0.2 years; maturity offset = -1.6 ± 0.7) or an active control group (CG) (n = 14; age = 11.8 ± 0.4 years; maturity offset = -2.51 ± 0.61). The training intervention lasted eight-week and was conducted during the in-season period, with twice-weekly sessions. Physical fitness tests were conducted before and after the intervention, including the 505 change-of-direction (CoD; [505 CoD test]), countermovement-jump (CMJ) height, standing-long-jump (SLJ) distance, and single-leg hop test for distance with dominant (SHTD-D) and non-dominant legs (SHTD-ND). A jump-based asymmetry score was calculated as the difference between HTD and HTND.

**Results:**

ANCOVA analysis revealed significant between-group differences in all physical fitness measures at post-test. Specifically, the PJT group showed significant large improvements in CMJ height, SLJ distance, HTD and HTND, and CoD speed (*d* = 0.84 to 2.00; ∆1.05% to 16.85%). Moreover, the PJT group showed a significant, small reduction in the inter-limb asymmetry score (*d* = 0.43; ∆-45.21%). In contrast, no significant changes were reported in the CG between pre-and post-tests (*d* = 0.07 to 0.24; ∆0.21% to 0.98%).

**Conclusions:**

The incorporation of PJT into the training schedules of prepubertal male soccer players resulted in positive effects on various measures of physical fitness. Furthermore, our findings suggest that PJT can reduce lower-limb asymmetry, which could potentially decrease the risk of lower limb injuries.

**Trial registration:**

This study does not report results related to healthcare interventions using human participants and therefore it was not prospectively registered.

**Supplementary Information:**

The online version contains supplementary material available at 10.1186/s13102-024-00821-9.

## Background

Soccer (also called football) is a team sport that places considerable demands on players’ physical fitness, including muscle power, sprint speed, and change of direction [CoD] speed, all of which are critical for optimal performance [1]. During both training and matches, soccer players must perform a wide range of activities, such as jumping, kicking, accelerating, and decelerating, sprinting, and changing directions at different angles numerous times [2]. There is evidence that sprint speed, jump height, and CoD speed can differentiate between soccer players of varying skill levels [3], underscoring the utility of these physical fitness factors for talent identification purposes [4]. Additionally, these same fitness components appear to correlate with a team’s final ranking in the league during a competitive season [5]. While growth and maturation cause improvements in these key components of physical fitness [6], the development of these qualities can be further optimized using targeted training interventions [7]. In fact, regular engagement in physical fitness interventions at an early age is useful to guide effective long-term physical fitness development, thereby enhancing the prospects for success in the later stages of an athlete's career [8, 9].

Additionally, some of the aforementioned qualities are performed unilaterally, which might lead players to develop a preference for using one limb over the other [3, 5]. Previous research has shown a significant correlation between inter-limb asymmetry derived from the drop jump test and slower sprint (10m: *r* = 0.52; 30m: *r* = 0.58) and CoD speed (*r* = 0.52 to 0.66) performance in female soccer players aged 21 years [10]. Likewise, Maloney et al. [11] reported an association between drop jump height asymmetries and slower CoD performance (*r* = 0.60) in healthy recreationally active males aged 22 years. In youth male and female tennis players aged 16 years, larger asymmetries in the drop jump test were found to be associated with reduced CoD speed performance in both the dominant and non-dominant legs [12]. It is worth mentioning though that the association between inter-limb asymmetry and measures of physical fitness was not consistent. For instance, Bishop et al. [2019] revealed no significant relationship between asymmetry measured during jump tests and physical fitness tests (i.e., 30-m linear sprint, and CoD speed tests) in elite male under-23 academy soccer players [13]. The lack of consistent outcomes pertaining to the relationship between inter-limb asymmetry and key measures of physical fitness in the literature implies that further investigations would be needed to bring forth more insights. Furthermore, there is evidence that increased levels of inter-limb asymmetries may increase the risk of injury in male youth soccer players [14, 15]. Unlike cross-sectional studies, there is a lack of intervention studies examining the effects of specific training methods on lower-limb asymmetries, particularly in prepubertal male soccer players [16]. Therefore, future research is needed to better understand how to mitigate existing asymmetries in athletic populations [16].

Plyometric jump training (PJT) is a popular, cost-effective, and feasible training method that is effective in improving physical fitness, particularly strength- and power-related variables, in youth athletes [17]. Additionally, PJT has been demonstrated to decrease the incidence and severity of sports-related injuries in athletes [18]. Although there has been an increase in the number of PJT studies, including those involving youth athletes, there remains a necessity for further high-quality studies, particularly randomized controlled trials to establish robust evidence, especially in prepubertal male soccer players [15, 19].

To the best of our knowledge, no prior studies have investigated the effects of PJT on physical fitness measures and lower-limb jumping asymmetries in prepuberal male soccer players [16]. Therefore, the objective of this study was to assess the effects of PJT on physical fitness measures and lower-limb asymmetries in prepuberal male soccer players. We hypothesized that prepubertal male soccer players who participated in PJT would demonstrate greater improvements in physical fitness measures and reductions in lower-limb asymmetries when compared to an active control group [16].

## Methods

### Experimental approach to the problem

A two-group repeated measures experimental design was implemented to examine the effects of eight weeks of bi-weekly in-season PJT on various measures of physical fitness and inter-limb asymmetries in prepubertal male soccer players, compared to their counterparts who continued with their regular in-season (February, March) soccer-specific training routine. Following the CONSORT guidelines [20], highly-trained youth soccer players from the same Tunisian regional soccer team were randomly (blinded allocation) divided into a PJT group and an active control group (CG). Both groups participated in five training sessions per week. The PJT group replaced 20 to 25 min of low-intensity soccer drills with PJT drills, on Tuesdays and Thursdays, over 8 weeks. The PJT sessions were supervised by the fitness trainer of the team who was not blinded to the group allocation. After completing the PJT portion of the session, the players in the experimental group joined their control-group peers and completed the remainder of their regular soccer-specific training. Two familiarization sessions were conducted two weeks before the baseline testing to familiarize participants with the tests and type of jumps employed in the study. In addition, proper technique was ensured through verbal cues and demonstration by the researchers throughout the intervention. To limit stress on musculotendinous unit, training volume was progressively increased. Pre- and post-training assessments included the tests countermovement jump (CMJ), standing long jump (SLJ), single leg hop with dominant (HTD) and non-dominant (HTND) leg, and CoD ability (i.e., 505 CoD test). All test measurements were conducted by the same assessors, and gathered data was later analyzed by the first author. The assessors were blinded regarding the allocation of participants to the CG and PJT groups. The warm-up protocol preceding testing included 5 min of submaximal running with CoD exercises, 10 min of submaximal plyometrics (20 verticals [i.e., CMJs] and 10 horizontal jumps [i.e., bilateral ankle hops]), dynamic stretching exercises, and 5 min of a sprint-specific warm-up. All tests were performed at least 48 h after the last training session or match, in the morning between 7:30 and 9:30 a.m., and under consistent environmental conditions (20–22°C, no wind). Three weeks prior to the start of the study, all participants performed twice weekly strength endurance exercises for muscles of the upper and lower limbs and the trunk using their own body mass to get prepared for the subsequent PJT program. The strength endurance training program included abdominal curls, back extensions, and squats. Participating athletes completed up to 5 sets of 15 repetitions each with 30 s rest in-between sets [21, 22].

### Participants

**Fig. 1:**
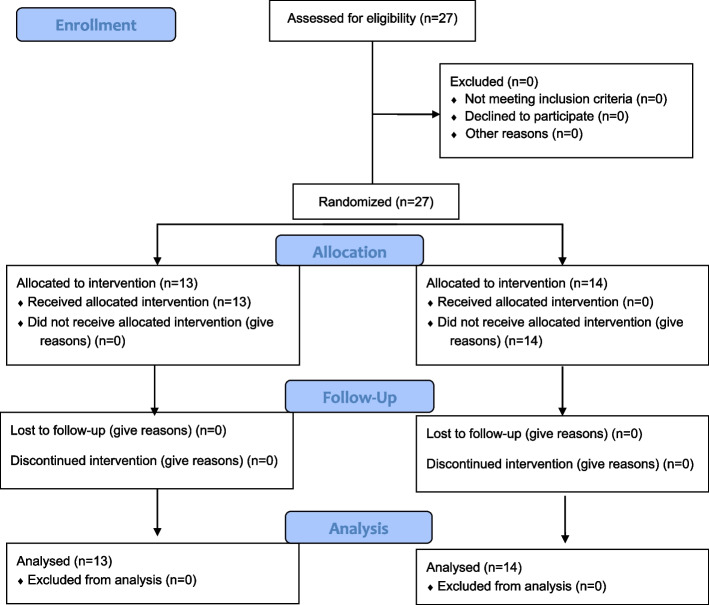
The diagram (The CONSORT: Consolidated Standards of Reporting Trials) includes detailed information on the interventions received

Figure 1 displays a CONSORT diagram of the levels of reporting and participant flow for the study.

We performed an a priori sample size calculation for the 10-m CoD speed test, contrasting intervention and control groups. We set the type I error rate at 0.05 and the statistical power at 80%. The estimated effect size of Cohen’s d = 0.96 is based on a randomized controlled trial by Sammoud et al. [23]. The analysis indicated that 12 participants per group would represent a sufficient sample. Therefore, the required number of participants was determined to be 24. To account for potential participant attrition twenty-seven prepubertal male soccer players were randomly allocated to a PJT group (n = 13; age = 12.7 ± 0.2 years; maturity offset = -1.6 ± 0.6 years) or an active CG (*n* = 14; age = 11.8 ± 0.4 years; maturity offset = -2.5 ± 0.6 years). All participants were classified as experienced players with 5.0 ± 1.1 years of systematic soccer training background comprising three to five training sessions per week. Further, it's important to note that all participants were in a healthy condition, without any history of moderate to severe musculotendinous injuries in the six months prior to the study commencement. Players who missed more than 20% of the total training session and/or more than 2 consecutive sessions were excluded from the study. The anthropometric data of both groups are presented in Table 1. The maturity offset method was used to estimate the maturity status of participants [24]. The applied equation was: Maturity offset = -9.236 + (0.0002708·leg length and sitting height interaction) – (0.001663·age and leg length interaction) + (0.007216·age and sitting height interaction) + (0.02292·weight by height ratio*100).

**Table 1:** Anthropometric characteristics of participants

	PJT group (*n* = 13)	Control group (*n* = 14)	
Age (years)	12.7 ± 0.2	11.8 ± 0.4*	
Height (m)	155.8 ± 7.4	148.1 ± 7.3*	
Body mass (kg)	47.9 ± 7.3	39.4 ± 5.3*	
Maturity offset (years)	-1.6 ± 0.6	-2.5 ± 0.6*	
APHV (years)	14.3 ± 0.5	14.3 ± 0.5 NS	

Data are presented as means ± standard deviations. *PJT* Plyometric jump training, *APHV* Age at peak height velocity

NS: denotes no significant difference (*p* > 0.05)

^*^denotes significant difference (*p*<0.05)

The present study was conducted in accordance with the latest version of the Declaration of Helsinki and protocol is in line with the standards for ethics in sport and exercise science research [25]. Before experimental testing, the protocol was approved by the local institutional review board of the Higher Institute of Sports and Physical Education of Ksar Saïd, Tunisia. Written informed consent was obtained from the parents/legal representatives of all participants before the commencement of the study.

### Anthropometric measures

Prior to the pretest assessment, a qualified anthropometrist, trained in accordance with the standardized procedures of the International Society for the Advancement of Kinanthropometry (ISAK), conducted all anthropometric measurements [26].

### Physical fitness tests

#### Countermovement jump

For the CMJ, participants started from an upright standing position and performed a fast downward movement by flexing the knees and hips before rapidly extending the legs and performing a maximal vertical jump. During the test, participants were instructed to maintain their arms akimbo. Jump height was recorded via an optoelectric system (Optojump, Microgate, SRL, Bolzano, Italy). A rest period of 1-min was allowed between trials. The best out of three trials was retained for further analysis. The between-trials intraclass correlation coefficient (ICC_3,1_) was 0.93.

#### Standing long jump

During the SLJ test, participants positioned themselves with their feet shoulder-width apart, standing in front of a designated starting line. They initiated the jump by swiftly flexing their legs and simultaneously moving their arms downward, aiming to jump as far as possible in a horizontal direction. When landing, participants were required to touch the ground with both feet simultaneously and maintain balance without falling forward or backward. The horizontal distance between the starting line and the heel of the rear foot was measured using a tape measure, with measurements recorded to the nearest 1 cm. Each participant performed three trials, and the best distance achieved among the three attempts was selected for further analysis. The reliability of the measurements between trials was assessed using the ICC, which yielded a value of 0.97, indicating a high level of consistency between the trials.

### Single leg hop test for distance with dominant and non-dominant leg

The single leg hop test for distance for the dominant leg (SHTD-D) and non-dominant leg (SHTD-ND) was conducted while participants adopted a standing position on the designated testing leg, with their hands on hips and their toes behind the starting line. Subjects were then instructed to hop forward as far as possible and land on the same leg. Upon landing, participants were required to ‘hold and stick’ their position for ~ 2 s. The leg used to kick a soccer ball is identified as the dominant leg [27]. The best out of three valid trials was recorded for further analysis. The between-trials ICCs were 0.94 and 0.91 for SHTD-D and SHTD-ND, respectively.

### The 505 change of direction test

In the 505 CoD test, the players positioned themselves in a standing stance, starting from a point 10 m away from the designated start line. Upon the start signal, they sprinted as quickly as possible through the start/finish line. At the 15-m mark, indicated by a cone marker, the players executed a 180° pivot and immediately returned to the start line, aiming to complete the test in the shortest time possible [28]. To ensure proper execution of the test, a researcher was positioned at the turning line. If a participant changed direction before reaching the designated turning point or turned off the incorrect foot, the trial was disregarded, and the participant was allowed to reattempt the trial after a recovery period. A rest period of three minutes was provided between each trial to minimize fatigue. Two trials were conducted, and the best performance achieved among the two attempts was selected for further analysis. The reliability of the measurements between trials was assessed using the ICC, which yielded a value of 0.90, indicating a high level of consistency between the trials.

### Plyometric jump training

The PJT program was implemented during the second half of the in-season period, specifically in February and March of 2022. For both groups, a standardized warm-up lasting 8 to 12 min was conducted prior to each training session. This warm-up included activities such as low-intensity running, coordination exercises, dynamic movements (e.g., lunges, skips), sprints, and dynamic stretching targeting the muscles of the lower limbs. At the start of each training week, the first PJT session was scheduled at least 48 h after the last soccer match that took place over the weekend. The second PJT session was conducted 72 h after the first session, specifically on Tuesdays and Thursdays. The PJT drills were incorporated at the beginning of the regular soccer training sessions. The specific details of the PJT protocol can be found in the attached table (Table 2). The total ground contacts per week gradually increased from 50 during the first week to 120 during the last week of training [29, 30]. Each PJT session included vertical (i.e., CMJs) and horizontal (i.e., two-footed ankles hop forward and double leg zigzag) jumps performed at a maximal intensity (i.e., maximal height and forward distance with a minimum contact time for vertical and horizontal jumping, respectively). During the first four weeks, all vertical and horizontal jumping was executed bilaterally whereas, during the last four weeks, horizontal jumping was performed bilaterally and unilaterally [29]. The same order of the PJT exercises was maintained during the 8 weeks: CMJ, CMJ-akimbo, two footed ankles hop forward and double leg zigzag jumps. In addition, as indicated in Table 1, the number of foot ground contacts fluctuated within the training program, ranging from 50 to 120 contacts per session, thereby affecting the number of sets and repetitions per set. To allow sufficient rest, a 90-s was provided between each set of exercises.

**Table 2:** Characteristics of the plyometric jump training program

Week	N° of sets (repetitions per set)	N° of total foot ground contacts		Type and order of jumps^a^
		Session 1	Session 2	
1	5–6 (10)	50	60	1. CMJ 2. CMJ with arms akimbo 3. Two footed ankles hop forward 4. double leg zigzag jumps
2	5–6 (10)	50	60	
3	5–6 (12)	60	72	
4	5–6 (12)	60	72	
5	5–6 (15)	75	90	
6	6–7 (15)	90	105	
7	7 (15)	100	100	
8	8 (15)	120	120	

^a^the order of the exercices was the same throughout the whole duration of the plyometric jump training program; *CMJ* Countermovement jump

### Soccer training protocol

The active CG participated in a regular soccer-specific training program over the 8-week intervention period with 5 training sessions per week lasting between 80 and 90 min. The PJT group participated in 3 soccer-specific training sessions per week that were similar to those of the CG, and the 2 PJT sessions substituted 2 soccer-specific training sessions so that the overall exposition time to training was identical between the 2 groups. In general, soccer training included training in fast footwork, technical skills and moves, position games, and tactical games.

### Statistical analyses

Data were tested for normal distribution using the Shapiro–Wilk test. Training effects were evaluated using an ANCOVA statistical model with baseline measurements entered as covariates. Additionally, effect sizes (ES) were determined by converting partial eta-squared from the ANCOVA output to Cohen’s *d*. To evaluate within-group pre-to-post performance changes, paired sample t-tests were applied. The ESs were determined from means, standard deviations, and correlation coefficients. According to Cohen [31], ES can be classified as small (0.00 ≤ d ≤ 0.49), medium (0.50 ≤ d ≤ 0.79), and large (d ≥ 0.80). Test–retest reliability was assessed using the ICC_(3, 1)_. Mean inter-limb asymmetry was calculated as a percentage difference between limbs in the unilateral tests using the following equation: (100 / [maximum value] × [minimum value] × -1 + 100) [32]. Data are presented as group means values and standard deviation for the pre-test and adjusted means and standard deviation for the post-test. The level of significance was set at *p* ≤ 0.05. Statistical calculations were conducted using SPSS 20.0 (SPSS Inc., Chicago, IL, USA).

## Results

All the players successfully completed more than 85% of the training sessions and were consequently fully included in the final analyses. The results presented in this section are included in Table 3. Significant between groups differences were detected for the chronological age, height, body mass, and maturity offset. Nevertheless, the maturity offset method indicated that all the participants were prepubertal (Table 1). In terms of physical fitness measures, no significant baseline differences between groups were observed (*p* > 0.05), except for the inter-limb asymmetry score (*p* < 0.05).

**Table 3:** Group-specific baseline and posttest performances after 8 weeks of in-season plyometric jump training on components of physical fitness in prepuberal soccer players

	**Pre**						**Post**					
	**PJTG**		**CG**				**PJTG**		**CG**			
	**M**	**SD**	**M**	**SD**	**Diff (95% CI)**	***Independent sample t-test p*-value**	**M**	**SD**	**M**	**SD**	**Diff (95% CI)**	***ANCOVA*** ***p*-value (Cohen’s *d*)**
CMJ (cm)	25.97	3.99	26.33	4.62	-0.36 (-3.79 to 3.07)	0.83	30.52	0.61	26.36	0.58	4.16 (2.41 to 5.91)	< 0.0001 (2.00)
SLJ (cm)	169.3 1	15.04	174.4 3	16.36	-5.12 (-17.60 to 7.36)	0.40	187.6 0	2.82	174.5 0	2.71	13.09 (4.95 to 21.23)	< 0.01 (1.36)
SHTD-D (cm)	161.9 2	13.57	159.8 6	13.06	2.06 (-9.42 to 13.55)	0.71	176.4 2	2.98	161.3 8	2.87	15.03 (6.46 to 23.61)	0.001 (1.48)
SHTD-ND (cm)	167.6 9	12.47	162.6 4	12.42	4.74 (-5.08 to 14.56)	0.33	177.5 2	2.05	163.8 7	1.97	13.65 (7.69 to 19.61)	0.001 (1.93)
505 CoD (s)	2.60	0.14	2.68	0.11	-0.07 (-0.16 to 0.02)	0.13	2.46	0.04	2.64	0.03	-0.18 (-0.29 to -0.06)	< 0.01 (1.30)
Asymmetry score (%)	8.11	12.47	4.72	2.75	4.15 (0.80 to 7.50)	0.01	3.36	1.10	6.68	1.06	3.32 (-6.68 to 0.27)	< 0.05 (0.84)

*M* mean, *SD* standard deviation, PJTG plyometric jump training group; CG control group; *CMJ* countermovement jump, *SLJ* standing long jump, hop test distance for the non-dominant leg, *CoD* change of direction speed test. *SHTD-D* single hop test for distance for the dominant leg; SHTD-ND single hop test for distance for the non-dominant leg; CI confidence interval

A significant large between-group difference at post-test was observed for CMJ height (*p* < 0.001; d = 2.00), SLJ (*p* < 0.01; d = 1.36), SHTD-D (*p* < 0.001; d = 1.40), SHTD-D (*p* < 0.001; d = 1.93), and 505 CoD (*p* < 0.01; *d* = 1.30) tests. More specifically, the PJT group achieved significant large pre-to-post training improvements in CMJ height (∆16.85%; d = 3.47), SLJ (∆9.77%; d = 1.15), SHTD-D (∆10.87%; d = 1.44), SHTD-ND (∆6.83%; d = 1.20), and 505 CoD (∆-6.53%, *d* = 1.06) tests. However, for the CG, no significant pre-to-post changes were found for CMJ height (∆0.76%; d = 0.07), SLJ (∆0.98%d = 0.24), SHTD-D (∆0.67%d = 0.08), SHTD-ND (∆0.35%d = 0.12), and 505 CoD (∆-0.21%, *d* = 0.08) tests.

Regarding the asymmetry scores, the ANCOVA analysis indicated a significant large between-group difference at post-test (*p* = 0.05, *d* = 0.84). A significant small pre-to-post reduction was found for the PJT group (change = -4.75%%, *d* = 0.43) but not for the CG (change = 1.96%, *d* = 0.33).

## Discussion

This study aimed to examine the effects of PJT on physical fitness measures and lower-limb asymmetry in prepubertal male soccer players. The main findings revealed significant moderate-to-large effects of PJT on CMJ, SLJ, HTD, HTND, and CoD performances. Additionally, the results suggested a small but significant reduction in the lower limb asymmetry index after training. Conversely, no significant changes between pre and post-tests were observed in the CG.

### Jumping ability

It is widely recognized that modern soccer demands a high level of jumping performance to enable soccer players to effectively cope with the elevated physical demands of a soccer match [2]. Jump performance is a valid indicator of talent identification, which can distinguish between elite and non-elite youth soccer players. Our statistical analyses revealed significant, large pre-to-post training improvements in jumping height performance (d = 3.47, d = 1.15, 1.44, and 1.20 for the CMJ, SLJT, HTD, and HTND, respectively) after the PJT program. In contrast, no significant pre-to-post changes were detected in the CG. Consistent with our findings, Ramirez-Campillo et al. [33] reported an improvement in CMJ height of 4.3% after eight weeks of PJT (ES = 0.2) in youth male soccer players aged 13 years. Furthermore, a systematic review with meta-analysis by Moran et al. [17] reported a moderate positive effect of PJT (d = 0.91) on jumping performance in prepubertal male soccer players. Although no mechanistic measures were conducted in this study, it is likely that the enhancement of the two essential components of the stretch–shortening cycle (SSC) after the PJT program contributed to the gains in jumping performance. Specifically, improvements in jumping ability after PJT are thought to be due to neural adaptations in terms of increased motor unit recruitment (i.e., intramuscular coordination) and better synergistic and less antagonistic muscle activation strategies (i.e., intermuscular coordination). These are potential mechanisms proposed to result in enhanced jumping performance after PJT [34]. However, other potential mechanisms, such as musculotendinous tissue adaptations (e.g., increased stiffness that can impact the muscle’s ability to generate force rapidly and increased cross-sectional area of the muscle) may also contribute to the improvement in jumping performance after PJT in youth soccer players [35, 36].

### Change of direction

CoD ability is a distinctive feature of performance in male soccer players [37]. Our results demonstrated a large pre-to-post improvement (∆-6.53%, *d* = 1.06) in CoD after PJT. In contrast, no significant pre-to-post changes were detected in the CG (*d* = 0.08). These results support those of previous studies such as Negra et al. [18] who reported a large improvement (d = 1.52) in CoD speed performance (i.e., Illinois agility test) after eight weeks of PJT in prepubertal male soccer players. Similarly, Bouguezzi et al. [29] revealed large improvements (d = 0.94) in the modified Illinois CoD performance test after an 8-week PJT program in prepubertal male soccer players. A recently published umbrella review [38] also indicated that PJT benefits CoD speed performance. The improvement in CoD performance may be attributed to enhanced SSC efficiency (i.e., rapid switch from eccentric to concentric muscle action), which is an important prerequisite to improve CoD movements [39]. Eccentric strength is a key factor for effective deceleration during CoD tasks [38]. In this sense, it seems that PJT contributed to better eccentric strength. This has probably occurred due to the increased eccentric overload during the descending and braking phases of PJT [40, 41]. The increase in eccentric strength of the knee extensors may have translated to a more effective ability to change direction. Additionally, the improvement in CoD performance may be related to the augmentation of the rate of force development through increased intermuscular and intramuscular coordination [34, 42], which is a preeminent factor in optimizing CoD performance [39]. Granacher et al. [43] also suggested that PJT can decrease ground reaction times through increased muscular force output and movement efficiency, which positively affect CoD performance. Further, Hakkinen et al. [42] reported that the improvement in CoD performance is moderated by neural factors such as higher levels of motor unit recruitment/synchronization and increased firing frequency, both of which can be improved after PJT [44].

### Asymmetry score

In soccer, inter-limb asymmetry is a potential risk factor for injury and can negatively impact performance [45]. Our results indicated a small but significant reduction in inter-limb asymmetry score for the PJT group (change = -4.75%, *d* = 0.43) after the intervention period. In contrast, the CG showed a worsening of the asymmetry score (change = 1.96%, *d* = 0.33), although this change did not reach statistical significance. These results are not surprising given that soccer is characterised by a high prevalence of unilateral movements, such as jumping, kicking, and CoD speed jumping, which can lead players to rely on one limb over the other. Therefore, incorporating PJT into training routines may be a useful strategy to mitigate inter-limb asymmetry and reduce the risk of injury in soccer players by reducing.

In recent years, studies have investigated the effects of various training interventions on inter-limb asymmetry across different sports, with inconsistent findings being reported [46–49]. For example, while Parados Mainer et al. [49] revealed a reduction in inter-limb asymmetry (ES: -0.76 to 0.49) following a 10-week additional injury prevention programme (FIFA 11 +) in adolescent female soccer players aged 13 years, another study by the same authors [49] showed no significant improvement in inter-limb asymmetry (ES = 0.26) after an 8-week combined strength and power training program in adolescent female soccer players aged 16 years. Similarly, Gonzalo Skok et al. [47] found a decrease in inter-limb asymmetry in young male soccer players aged 16 years following three different unilateral strength training strategies. Similar to the findings of Pardos-Mainer et al. [48], the change in inter-limb asymmetry in our study was small. It is important to note that asymmetry is a ratio metric and can result in large SD values relative to the mean (as seen in Table 3). Thus, a large standard deviation can often prevent statistical significance from being achieved in asymmetry analysis, which has been observed in previous studies on this topic [50, 51]. This may be a contributing factor to the inconsistent findings reported in the literature. Our study showed a significant reduction in inter-limb asymmetry score among prepubertal male soccer players after undergoing an 8-week PJT program, although the reduction was small in magnitude. This could be attributed to the balanced load between limbs in both bilateral and unilateral vertical and horizontal jumps that were included in the PJT program, which may have helped to mitigate the neuromuscular deficit of the weaker side. As such, this resulted in a reduction in the overall level of asymmetry. It is important to note, however, that future studies with longer training periods are needed to confirm these findings and to gain a better understanding of the impact of PJT on inter-limb asymmetry in young soccer players.

### Strengths and limitations

This study has strengths and limitations. In terms of the strengths, this is the first empirical investigation that examined the effect of PJT on inter-limb asymmetries in prepubertal male soccer players. In addition, this study was conducted during the in-season period of the year. Compared to interventions during the pre-season, the effects observed during the current study may be attributed with a greater degree of certainty to PJT intervention, rather than to a low level of basal physical fitness of the players at the beginning of a pre-season period. Moreover, PJT interventions were frequently incorporated alongside regular youth training [29, 30], posing a challenge in isolating the exclusive impact of the PJT intervention. To address this, the PJT in this study substituted 20 to 25 min of low-intensity soccer drills twice a week over an 8-week period. This substitution increases our confidence in attributing the observed effects primarily to PJT. With respect to the limitations, the primary drawback of this study is the lack of biomechanical and electrophysiological testing methods. This may limit the insights into the physiological adaptive mechanisms associated with physical fitness improvements and reduced asymmetry after PJT. As such, integrating such measures in future studies would be beneficial. Second, the asymmetry results obtained in this study are specific to the test used and can’t be generalised to other asymmetry protocols [16, 32]. It would have been beneficial to incorporate an additional test for quantifying asymmetry in future studies to assess the consistency of the findings. Third, while the current study indicated positive effects of PJT on asymmetry level, the magnitude of the effect was small, and future studies with larger sample sizes are needed to confirm these findings. *Fourth,* our results showed significant difference between the control and experimental groups for anthropometric and maturity offset variables. However, both groups were classified as prepubertal. Moreover, evidence from a recently published systematic with meta-analysis indicated that maturity status does not moderate the effects of plyometric training on measures of physical fitness. More particularly, authors reported similar plyometric training-related adaptation between pre- and post-pubertal youth in most outcomes, except CoD speed [52]. Nonetheless, future studies should consistently report maturity offset, particularly in the field of jump training, where most published studies have failed to provide a description for this potentially relevant moderator factor [19], including soccer studies. Finally, the current results are specific to prepubertal male soccer players and cannot be generalized to prepubertal females. As such, upcoming studies including female participants would be needed to extend the findings of this study, particularly in terms of asymmetry.

## Conclusions

The findings of the current study indicated moderate-to-large improvements in physical fitness measures in prepubertal male soccer players. Of note, results showed that 8 weeks of PJT contribute to reducing the level of inter-limb asymmetry in the same population. The decrease in inter-limb asymmetry may potentially mitigate the risk of lower limb injuries. These results extend the existing literature by highlighting the additional benefits of PJT in reducing inter-limb asymmetry in youth soccer players. Therefore, incorporating PJT into regular soccer training could be beneficial not only for improving physical fitness but also for reducing the risk of injuries associated with asymmetry in prepubertal male soccer players. These findings may guide practitioners in designing effective PJT programs for this population.

### Supplementary Information

**Additional file 1.** CONSORT 2010 checklist of information to include when reporting a randomised trial_*_.

## Data Availability

The datasets generated and/or analysed during the current study are not publicly available. Upon request, the corresponding author will share the data set.

## Abbreviations

CoD: Change of direction
PJT: Plyometric jump training
CG: Control group
d: Cohens’d
CMJ: Countermovement jump
SLJ: Standing long jump
HTD: Single leg hop test for distance with dominant leg
HTND: Single leg hop test for distance with non-dominant leg
ES: Effect size
ICC: Intraclass correlation coefficient
ANCOVA: Analysis of covariance
